# Laser Treatment Monitoring with Reflectance Confocal Microscopy

**DOI:** 10.3390/medicina59061039

**Published:** 2023-05-28

**Authors:** Stefania Guida, Caterina Longo, Simone Amato, Anthony M. Rossi, Marco Manfredini, Silvana Ciardo, Marco Spadafora, Steven P. Nisticò, Santo R. Mercuri, Franco Rongioletti, Nicola Zerbinati, Giovanni Pellacani

**Affiliations:** 1 School of Medicine, Vita-Salute San Raffaele University, 20132 Milan, Italy; 2 Dermatology Clinic, IRCCS San Raffaele Hospital, 20132 Milan, Italy; 3 Centro Oncologico ad Alta Tecnologia Diagnostica, Azienda Unità Sanitaria Locale—IRCCS di Reggio Emilia, 42123 Reggio Emilia, Italy; caterina.longo@unimore.it (C.L.);; 4 Department of Dermatology, University of Modena and Reggio Emilia, 41124 Modena, Italy; marco.manfredini@unimore.it (M.M.); silvana.ciardo@unimore.it (S.C.); 5 Department of Health Sciences, Magna Graecia University, 88100 Catanzaro, Italy; simonamato94@gmail.com (S.A.);; 6 Department of Medicine, Dermatology Service, Memorial Sloan-Kettering Cancer Center, New York, NY 10021, USA; rossia@mskcc.org; 7 Department of Dermatology, Weill Cornell Medical College, New York, NY 10021, USA; 8 Clinical and Experimental Medicine PhD Program, University of Modena and Reggio Emilia, 41124 Modena, Italy; 9 Dermatology and Cosmetology Unit, IRCCS San Raffaele Hospital, 20132 Milan, Italy; 10 Department of Medicine and Surgery, University of Insubria, 21100 Varese, Italy; nicola.zerbinati@uninsubria.it; 11 Dermatology Unit, Department of Clinical Internal Anesthesiologic Cardiovascular Sciences, Sapienza University of Rome, 00185 Rome, Italy

**Keywords:** laser, reflectance confocal microscopy, laser monitoring, rejuvenation, scar, pigmentation

## Abstract

Laser treatments have become popular in Dermatology. In parallel to technologic development enabling the availability of different laser wavelengths, non-invasive skin imaging techniques, such as reflectance confocal microscopy (RCM), have been used to explore morphologic and qualitative skin characteristics. Specifically, RCM can be applied to cosmetically sensitive skin areas such as the face, without the need for skin biopsies. For these reasons, apart from its current use in skin cancer diagnosis, our systematic review reveals how RCM can be employed in the field of laser treatment monitoring, being particularly suitable for the evaluation of variations in epidermis and dermis, and pigmentary and vascular characteristics of the skin. This systematic review article aims to provide an overview on current applications of RCM laser treatment monitoring, while describing RCM features identified for different applications. Studies on human subjects treated with laser treatments, monitored with RCM, were included in the current systematic review. Five groups of treatments were identified and described: skin rejuvenation, scar tissue, pigmentary disorders, vascular disorders and other. Interestingly, RCM can assist treatments with lasers targeting all chromophores in the skin and exploiting laser induced optical breakdown. Treatment monitoring encompasses assessment at baseline and examination of changes after treatment, therefore revealing details in morphologic alterations underlying different skin conditions and mechanisms of actions of laser therapy, as well as objectify results after treatment.

## 1. Introduction

Over the past few decades, non-invasive treatments are increasingly requested in Dermatology [1,2,3,4]. Among these, laser therapy became popular due to technologic development leading both to the availability of different light wavelengths targeting different chromophores and to protocols reducing the downtime of treatment, according to patients’ requests [3,4,5,6].

In this scenario, in vivo reflectance confocal microscopy (RCM) has emerged as a non-invasive technique enabling horizontal visualization at different layers of the skin with good contrast and high resolution, providing cytologic and architectural details [7,8,9].

It has been proven as an excellent add-on tool for diagnostic purposes in Dermatology as well as for the analysis of healthy skin, since RCM provides an optical ‘‘histological’’ biopsy of the living tissue in a totally non-invasive manner, therefore avoiding scars, which is pivotal for aesthetic areas such as the face [10]. Specifically, RCM has been mainly employed in Cosmetic Dermatology to examine presence of regular/irregular keratinocytes at epidermis, collagen morphology and eventual elastosis at dermal level and pigmentary characteristics of the skin [8,10,11]. Importantly, these parameters have been recently standardized according to semi-quantitative and qualitative scales in order to improve recognition, reliability and reproducibility of evaluations [10]. Another advantage of non-invasive RCM is its ability to enable repeated examination of a given skin area, facilitating dynamic evaluation of skin changes, such as those that occur during treatment monitoring. RCM has been used to assess the effectiveness of various laser therapies in treating a variety of skin conditions, including acne, rosacea, and post-inflammatory hyperpigmentation. By offering non-invasive, real-time evaluation of skin changes, RCM can assist clinicians in customizing laser treatments to meet the individual needs of patients, optimizing treatment outcomes [10,12].

To summarize, RCM has become an essential tool in the field of dermatology, revolutionizing the approach of clinicians towards non-invasive skin treatments. In addition to its diagnostic and treatment monitoring applications, RCM has also been used to assess the efficacy of laser therapy in various skin conditions, including skin rejuventation, scars, pigmentatry and vascular disorders. By providing non-invasive, real-time evaluation of skin changes, RCM can assist clinicians in tailoring laser treatments to meet the unique needs of individual patients, resulting in optimal treatment outcomes.

Currently, an overview about RCM in laser treatment monitoring is lacking. Therefore, we systematically review literature on the topic in order to summarize fields of application and to identify pre- and post-treatment RCM features that can be assessed in laser treatment monitoring.

## 2. Materials and Methods

Studies conducted on human subjects involving laser treatments for skin conditions monitored with RCM were screened.

Electronic databases were systematically searched and included MEDLINE (PubMed), Web of Science and Cochrane library databases. Search strategy adopted was similar across the databases and developed using the following keyword: “laser” AND “reflectance confocal microscopy”. Our search included studies from inception to October 2022.

Two authors independently screened the abstracts for inclusion and exclusion criteria (SG and SA). In case of doubt or discordance, a third opinion was obtained (CL).

Studies were excluded based on the following criteria:

- language other than English
- in vitro or animal studies
- not involving laser treatments
- concerning tumors and not used to understand underlying mechanisms of laser therapies
- studies without specific RCM features described before and after treatment
- studies involving less than 8 patients

From each of the included studies, the following data were extracted: first author, year of publication, indication, number of patients and numbers of female patients, age, skin type/ethnicity, study type, laser type, RCM criteria at baseline and variations post-treatment, follow up timing, efficacy and safety.

## 3. Results

### 3.1. Study Selection

**Figure 1:**
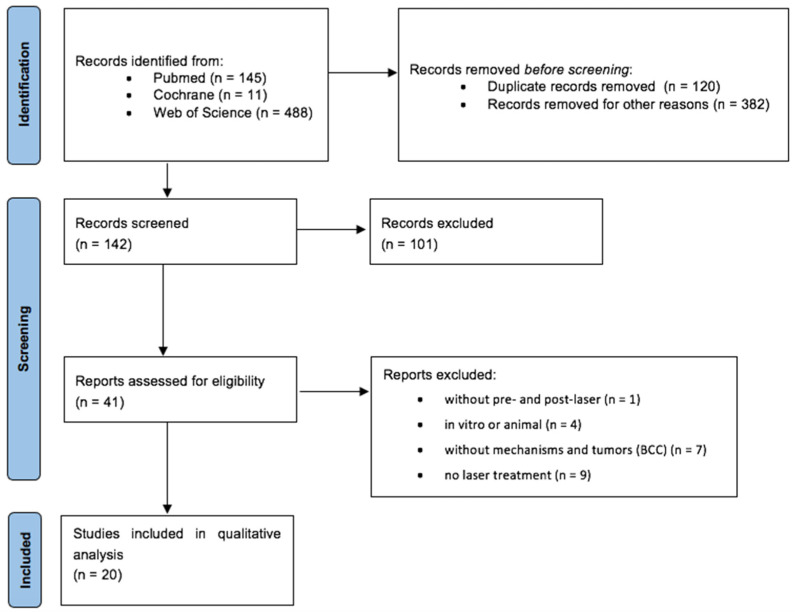
PRISMA diagram.

A total of 142 records were screened after duplicate and preliminary screening removal. Based on title/abstract screening, 101 studies were excluded. Forty-one full texts were thus assessed for eligibility and 20 studies were included in the qualitative synthesis, Figure 1.

Based on skin disease, 5 types of applications were identified: skin rejuvenation (n = 5), scar tissue (n = 6), pigmentary disorders (n = 5), vascular disorders (n = 2) and other (n = 2) [13,14,15,16,17,18,19,20,21,22,23,24,25,26,27,28,29,30,31,32]. Based on laser source employed in included studies, different laser sources were distinguished, those targeting water in the skin such as fractional C02 laser (n = 5) and erbium (n = 3), both ablative and non-ablative, those absorbed by hemoglobin, such as Nd:YAG nm (n = 1) and pulsed dye laser (n = 2). Additionally, laser wavelengths targeting pigment were also employed, including Q-switched laser (n = 5) and picosecond laser (PSL) (n = 4). Interestingly, PSL induce intraepidermal and dermal vacuole formation through laser-induced optical breakdown (LIOB), therefore resulting in multiphoton ionization due to high temperature and pressure created by high-energy irradiation with extremely short pulse durations, leading to additional applications.

A summary of RCM features is presented in Table 1; an overview of studies included is reported in Table 2.

### 3.2. Skin Rejuvenation

A total of 5 studies explored the role of RCM in laser monitoring for skin rejuvenation [13,14,15,16,17], Table 2. Most of the studies reported the use of lasers targeting water into the skin, including fractional CO_2_ laser and non-ablative fractional erbium laser while one study encompassed 1064 nm fractional PSL use, exploiting laser induced optical breakdown (LIOB). A total of 35 patients were treated for skin rejuvenation of the face while 18 for the neck. Mean follow up of patients was 1 to 4 months. Interestingly, RCM treatment monitoring enabled the visualization of variations supporting clinical improvement observed after laser treatments.

Early RCM parameters after laser treatment included the onset of micro-holes or microcolumns within the honeycombed pattern, corresponding to the micro-ablation induced by the fractional lasers, and dendritic cells at epidermal level, related to post-treatment inflammation, 1 to 6 weeks after treatment [14,15]. About 1 to 4 months after treatment, a clinical reduction of hyperpigmentation associated to aging (photoaging) was found to correspond to the reduction of both epidermal and dermo-epidermal junction (DEJ) RCM features of pigmentation, represented by mottled pigmentation or polycyclic papillary contours, respectively. Importantly, an increased number of dermal papillae and appearance of long straight fibers presenting a parallel alignment, or reticular collagen, was observed [13,14,15,16,17]. Interestingly, this last RCM feature corresponded to a clinical improvement of wrinkles/rhytids [16,17].

### 3.3. Scar Tissue

A total of six studies concerning treatment monitoring with RCM after laser treatments of scar tissue were included. Two studies involved acne scars, other two atrophic and hypertrophic surgical scars and the remnant two striae distensae. Similarly to skin rejuvenation, laser sources included CO_2_ fractional laser and non-ablative resurfacing lasers as well as PSL. RCM features included honeycombed or cobblestone pattern in epidermis, epidermal thickness, dermal papillae at DEJ and thin reticulated fibers or coarse collagen at dermal level [15,18,19,20,21,22], Table 2.

In detail, immediately after fractional CO_2_ laser or PSL to treat acne or surgical scars, black micro-holes surrounded by well-defined or fringed borders within the surrounding tissue could be observed [15,18,19,20,21,22]. Following the first week after treatment, a progressive repair of skin layers was observed. Three to 6 months post-treatment, thin reticulated collagen fibers arranged to form a net, with variable brightness of the collagen fibers, were observed [15,18,19,20]. Interestingly, this arrangement has been related to collagen remodeling [14].

**Figure 2:**
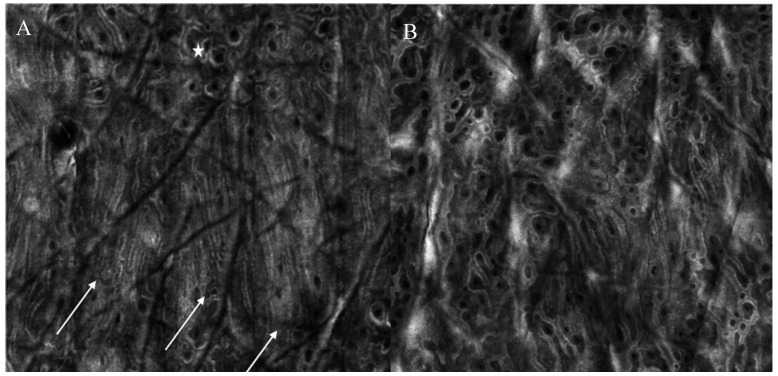
Reflectance confocal microscopy images of a patient with striae distensae at baseline and one month after 5 CO_2_ laser sessions. (**A**) At baseline, neat wall feature can be observed (white arrows), with hyper-reflective compact collagen and elongated papillae as well as the presence of an area of regular architecture (white star) (**B**) After treatment, neat-wall is not detectable and the architecture is predominantly composed of roundish papillae.

Interestingly, both surgical scars and striae distensae showed parallel collagen fibers, mainly orthogonal to major axis of scar tissue. These parallel collagen fibers were significantly reduced after CO_2_ laser and PSL for striae distensae [21,22]. Interestingly, a new confocal feature, the “neat wall”, was described at baseline in striae distensae [21], Figure 2. This parameter corresponds to a distortion of the normal DEJ, visible only in RCM mosaic images; it resembles a well-demarcated wall separating regular areas of DEJ, which are formed by round papillae and fibrillar or reticular collagen (definition tab RCM). This parameter was reduced in patients receiving more than 4 CO_2_ laser sessions, as compared to those receiving ≤4 sessions at 4-week post-treatment [21].

### 3.4. Pigmentary Disorders

Due to the ability of RCM to visualize pigmentation at different layers, RCM has been used as a treatment monitoring tool in different pigmentary disorders: solar lentigines, café au lait macules (CALMs), infraorbital dark circles, melasma [23,24,25,26,27], Table 2. Intuitively, the main laser source employed was Q-switched and a comparison with this laser source and PSL was available for one study.

In solar lentigines, edged dermal papillae and polycyclic papillary contours were observed at baseline. Immediately after Q-switched treatment, dark structureless areas could be observed throughout the epidermis and dermal papillae were hyporefractive. After 10 days, at the DEJ, non-edged dermal papillae were observed, in 9 out of 12 cases containing a few melanophages. Bright reflective rims surrounding the dermal papillae were no longer observed at the DEJ [23].

For CALMs, length and density of papillae were estimated. Interestingly, CALMs with irregular borders showed lower length and density of papillae as compared to those with smooth borders and better response to laser treatment [24].

Two studies were performed concerning the treatment of infraorbital dark circles in a total of 60 women of which half treated with Q-switched ruby and Q-switched Nd:YAG laser. At baseline, greater melanin deposition in the upper dermis of the dark circle area was observed, as compared to the cheekbone skin (control), but no significant difference in epidermal pigmentation. After 8 sessions, about 70% of subjects showed over 50% improvement of pigmentation, while the control area did not show any variations [25,26].

**Figure 3:**
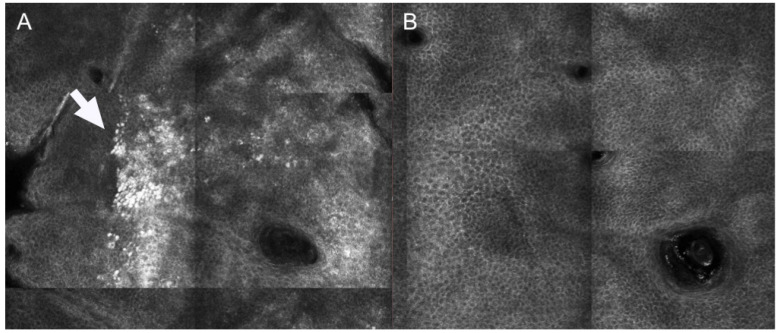
Reflectance confocal microscopy images of patient showing superficial melasma at baseline and 3 months after Q-switched laser treatment. (**A**) At baseline, mottled pigmentation (white arrow) can be observed at epidermal level (**B**) After treatment, RCM shows disappearance of mottled pigmentation and recover of regular honeycombed pattern.

Differently from what observed for dark circles, melasma patients can show pigmentation located at different layers. Jo et al. compared the effects of PSL and Q-switched laser on melasma. After treatment, either an increase of activated melanocytes at basal layer of epidermis or an increased amount of melanophages were observed at upper dermis 24 h after treatment [27]. Interestingly, the presence of dendritic-shaped cells after treatment has been associated with a relapse of melasma 3 months after multiple sessions of Q-switched laser treatment [28]. Figure 3 shows the presence of superficial pigmentation represented by mottled pigmentation at epidermal level at baseline and the disappearance of the pigmentation after Q-switched laser treatment.

### 3.5. Vascular Disorders

Treatment monitoring of port-wine stains (PWS) with RCM has been reported in two papers [29,30], Table 2. PWS are a type of vascular malformation that affect the skin and can cause significant psychological distress. Treatment options include pulsed dye laser (PDL) therapy, which targets the abnormal blood vessels to reduce their appearance. Based on different clinical response and RCM analysis of blood flow, RCM features associated to resistance to treatment for facial PWS were identified, being represented by high blood flow, large diameter, deep location [30]. Interestingly, based on different depths of vessels, different pulse durations of laser are related to different reduction of diameter vessels while no influence has been observed for density [29].

Overall, RCM is a valuable tool for monitoring the vascular response to laser therapy and for characterizing the microvascular changes associated with various vascular disorders. Further research is needed to fully understand the potential applications of RCM in the field of vascular medicine.

### 3.6. Other Applications

Laser treatment monitoring with RCM has also been explored in other fields [31,32], Table 2. Wound healing has been studied after CO_2_ laser on healthy skin and results observed were in line with previous findings related to applications for skin resurfacing and scar tissue treatment [31].

Additionally, RCM has been employed to monitor sebaceous hyperplasia treated with dye laser. At baseline, lesions appeared like dilated sebaceous duct surrounded by dilated vessels [32].

## 4. Discussion

Nowadays, laser treatment monitoring can be performed with non-invasive skin imaging techniques, such as RCM [14,15,16,17,18,19,20,21,22,23,24,25,26,27,28,29,30,31,32]. RCM is an advanced technique enabling the visualization of the skin at a quasi-histological resolution [7,9]. It has been mainly applied to the field of skin cancers [33,34] but has expanding applications in Cosmetic Dermatology [1,35]. As a matter of fact, the possibility to visualize pigmentation at different layers, collagen and vessels in a non-invasive manner, makes RCM particularly suitable to monitor laser treatments. Accordingly, when laser treatments are applied on cosmetically sensitive areas such as the face or are employed to treat aesthetic conditions, it would be preferable to avoid scarring as a consequence of skin biopsies [36,37].

Our results highlight that RCM can be applied to monitor skin rejuvenation treatments, scar tissue, pigmentation and vessels at different layers after laser therapy.

Specifically, RCM applied to skin rejuvenation and scar tissue treatment procedures revealed the advantage to monitor of skin healing process after laser therapy and to objectify variations of epidermis, DEJ and collagen or elastosis in the dermis. Additionally, RCM enables the visualization of micro-holes or microcolumns within the epidermal pattern, that have been associated to fractional laser treatment [14,15]. Similar findings were observed with histology [13].

Considering the high reflectivity of melanin, RCM is particularly useful to study pigmention distribution in different skin layers in different pigmentary disorders [38], overcoming the limitations related to other non-invasive tools, such as Wood’s lamp and dermoscopy which cannot locate the pigment precisely [39]. Interestingly, both pigment depth and the presence of dendritic-shaped cells in the epidermis have been associated with poor response to laser treatments, therefore highlighting the prognostic impact of RCM [27,28].

Our study reveals that specific terminology employed for healthy skin analysis can also be applied to define variations occurring after laser approach for skin rejuvenation, scar tissue and pigmentation [8,10,14,37].

Lastly, for vascular disorders, RCM has been employed to monitor the changes in blood vessels following laser treatments for port-wine stains. The ability to assess the depth, diameter, and density of blood vessels non-invasively may help optimize treatment parameters and improve overall treatment outcomes [29,30].

Currently, there are no studies comparing RCM with other techniques in laser treatment monitoring but some authors reported the alternative or complementary use of other techniques such as digital photography with automated features count and 3D imaging. Histologic studies, representing the reference for skin analysis, are mainly employed on ex vivo samples or in vivo in areas other than the face or on scars on the face in order to minimize visible scarring in aesthetic sites [40,41,42]. Therefore, many non-invasive techniques have been applied to the field of laser monitoring, apart from RCM, such as dermoscopy, digital photography with automatic features count (VISIA) and 3D assessment [3,43,44,45,46,47]. These techniques enable the visualization of pigment and vessels but, differently from RCM, they cannot be employed for the analysis of dermal features such as collagen characteristics and analyses per each skin layer.

In conclusion, RCM is a non-invasive technique that has shown promise in monitoring a variety of laser treatments in dermatology providing insights into the observation of variations associated with clinical improvement. RCM features identified in our systematic review can be applied to future studies to objectively evaluate changes in the skin and improve our understanding of the mechanisms of action of lasers. By facilitating non-invasive, real-time evaluation of skin changes, RCM can assist clinicians in tailoring laser treatments to meet the unique needs of individual patients, ultimately leading to optimal treatment outcomes.

## Figures and Tables

**Table 1:** Description of RCM features employed in laser treatment monitoring, classified according to skin layer.

	RCM Findings
*Epidermal layer*	
Regular honeycombed [14,18,19,23]	Polygonal keratinocytes with homogeneous size and shape; cell border is well outlined and preserved
Mottled pigmentation [14,16,17]	Clustered bright keratinocytes in context of honeycombed pattern
Spongiosis [18]	Honeycombed pattern where the outline of the epidermal cells and the intercellular junctions appeared brighter and larger compared with normal skin
Exocytosis [18]	Bright particles corresponding to lymphocytes
Fine scales [23]	Highly reflective round-to-polygonal areas
Micro-thermal zone or micro-injuries-ablative zone [14,18,19,31]	empty spaces or black micro-holes
Micro-thermal zone -thermal modified zone [31]	areas with a whitish ring exhibiting high reflectance
Extracellular deposits of melanin [23]	bright round-to-polygonal areas and aggregated granules were observed throughout the epidermis
*Dermo-epidermal junction*	
Polycyclic papillary contours [14,17]	Bulbous projections and cords, with variably convoluted arrangement
Edged dermal papillae [23]	Dark round-to-oval structures surrounded by a rim of bright monomorphic cells
Sebaceous glands [31]	Roundish to oval-shaped annular structures, corresponding to glandular parenchyma and centered by hair follicles
*Dermis*	
Thin reticular collagen [16,21]	Bright thin fibrillar structures forming delicate weblike pattern; this structure can be detected around follicular openings
Coarse collagen [14,16,18,20]	Coarse filamentous thick structures with tendency to be packed; weblike pattern is still observable but with larger and irregularly spaced meshes
Huddle collagen [14]	Large hyporefractive blotches of amorphous and hyporeflective material; individual collagen fibers are no longer visible
Curled bright fibers [14]	Highly refractive thick and short undulated fibers, sometimes forming compact masses when severe solar elastosis is present
“Neat-wall” [21]	distortion of the normal DEJ, visible only in mosaic images, similar to a well- demarcated wall separating regular areas of junction, which are formed by round papillae and fibrillar or reticular collagen

**Table 2:** Summary of characteristics of included studies.

Author, Date	Indication	N	Females, n (%)	Age, Mean ± SD (Range)	Skin Type/ Ethnicity	Study Type	Laser Type	RCM Criteria at Baseline	RCM Variations Post-Treatment	Follow Up Timing	Efficacy	Safety	Notes
Skin rejuvenation													
Shin, 2013 [13]	Rejuvenation	11	100%	26.8 ± 2.8 years (range from 24 to 30)	skin type III–IV	Retro	1550 nm erbium glass fractional laser	Normal dermal papillae, active dermal papillae, and melanized dermal papillae	At 4 weeks after treatment, the total number of dermal papillae showed a significant increase, compared with pretreatment (*p* = 0.01), while no difference was found for “active” and “melanized” papillae	up to 4 weeks	at 4 weeks, skin roughness was decreased, and net elasticity (R5) increased; however, these findings did not reach significance.	No serious adverse events	Melanized dermal papillae’ was defined as more than 50% of the bright shining circles of dermal papillae ‘Active dermal papillae’ when dermal papillae had red blood cells (RBC) in the capillary loop.
Longo, 2013 [14]	skin rejuvenation	11 (1 excluded)	-	aged 52–69	-	Retro	ablative fractional CO_2_ laser+ radiofrequency	Irregular Honeycombed pattern n = 10 (100%) mottled pigmentation n = 5 (50%) Dendritic-shaped cells Polyciclic Papillary contours n = 4 (40%) coarse collagen n = 10 (100%) coarse + huddle n = 2 (20%) prevalent huddle n = 5 (50%) curled bright fibers + huddle collagen n = 2 (20%)	At 3-week, micro-holes within the honeycombed pattern, dendritic cells at epidermis clearance of mottled pigmentation and polycyclic papillary contours, substitution of huddle collagen with coarse collagen and appearance of long straight fibers presenting a parallel alignment At 6 and 12 weeks, disappearance of dendritic cells at epidermis and persistence of other features observed at 3-week.	Up to 3 months	complete disappearance of the mottled pigmentation. Improvement the skin color. visible re-epithelization of micro-holes. collagen remodeling: long straight fibers with parallel arrangement	No relevant side effects were recorded at any time.	
Cameli, 2014 [15]	Rejuvenation and atrophic acne scars	10 (4 photoaging)	70%	28 to 55 (mean age 39.2)	Fitzpatrick II and III	Retro	Fractional Laser with Fractional Laser Plus Radiofrequency	No inflammation Evaluation of dermal fibers.	With regard to the inflammatory effect of laser treatment, infiltrated and dilated vessels in the upper dermis and microscopic signs of inflammation were assessed using RCM monitoring of the presence of inflammatory cells. After 1 week, in the site of laser plus radiofrequency treatment, microcolumns had completely disappeared, as had any features of inflammation or vasodilatation around the spot. Improvement of dermal fibers was also evident with RCM	Up to 3 months	In terms of skin aging, the results evaluated by the physician were better on the side of the skin treated using fractional laser + radiofrequency (excellent 75%, good 25%, sufficient 0%) and followed by more rapid healing than the skin treated with fractional laser only (excellent 25%, good 75%, sufficient 0%). Patient evaluation showed the same trend	Prolonged burning sensation above all on the laser + radiofrequency side, erythema and edema more on the laser side	
Bencini, 2015 [16]	Neck rejuvenation	18	100%	50.1 + 4.6 (range 44–59)	-	Retro	non-ablative fractional 1540 erbium glass laser	parallel microwrinkles mottled pigmentation, different collagen types	reduction of number of parallel micro-wrinkles, reduction of mottled pigmentation, increase of reticular collagen and reduction of coarse collagen	1 month 3 months	Significan improvement of dyschromia and superficial wrinkles, only slight improvement in deeper horizontal folds; no change in skin laxity Based on the 6-point grading scale, respectively.	Edema and erythema rapidly disappeared	
Guida, 2021 [17]	Photoaging	10	70%	>45 years 58.2 ± 5.9	-	Retro	1064 nm Nd:YAG PSL	Mottled pigmentation n = 10 (100%), Polyciclic papillary contours n = 7 (70%) Long bright collagen fibers n = 0	Significant reduction of mottled pigmentation n = 2 (20%), *p* = 0.001, reduction of polyciclic papillary contours n = 3 (30%), and increase in long bright collagen fibers n = 4 (40%)	4 months	A significant reduction of clinical parameter of dyscromia and wrinkles and rhytids parameters has been observed on the decolletage and face.	local and self-limiting adverse events	
Scar tissue													
Bencini, 2012 [18]	Acne scars	87	58%	29 years (range: 20–45)	-	Retro	1540 nm Erbium laser	Baseline: bright and grossly arranged coarse collagen regular honeycombed pattern.	30 mis after laser session: Spongiosis, exocytosis, well-defined large deposits of homogeneously reflective material were observed at dermal level (corresponding to micro-thermal zones) At 3-month, complete recovery of the honeycombed pattern, thin reticulated collagen fibers arranged to form a net, variable brightness of the collagen fibers distributed within a lesion that has been related to the increased collagen remodeling by fibroblasts.	3 weeks 6 months	Improvement was assessed according to a clinical scale. At 1-month 89% of patients had marked improvement. At 3-month, a total of 45% patients referred an improvement >50%, 39% an improvement of 21–50%, and 16% an improvement <20%.	In all patients, post-laser erythema and edema persisting for 1–4 days occurred. In 4/87 (5%) patients, a mild acneiform eruption appeared 2–3 days after one or more laser sessions and it was well controlled by topical clindamycin. In 1/87 patient (Fitzpatrick skin type V), a slight hyperpigmentation developed and faded within 1 month.	replacement of coarse collagen with a new one, similar to the collagen seen in healthy skin
Cameli 2014 [15]	Rejuvenation and atrophic acne scars	10 (6 acne scars)	70%	28 to 55 (mean age 39.2)	Fitzpatrick II and III	Retro	CO_2_ Fractional Laser with Fractional Laser Plus Radiofrequency–single session	No inflammation Evaluation of dermal fibers.	With regard to the inflammatory effect of laser treatment, infiltrated and dilated vessels in the upper dermis and microscopic signs of inflammation were assessed using RCM monitoring of the presence of inflammatory cells. One week after treatment, complete physiologic healing of the tissues was clinically and confocally evident. After 1 week, in the site of laser plus radiofrequency treatment, microcolumns had completely disappeared, as had any features of inflammation or vasodilatation around the spot. Improvement of dermal fibers was also evident with RCM.	Up to 3 months	clinical improvement of the boxcar and rolling scars in terms of depth and tissue remodelling that was more evident on the side of the face treated using fractional laser plus radiofrequency (excellent 50%, good 50%, sufficient 0%) than on the side treated with laser alone (30% excellent, 40% good, 30% sufficient) Healing was faster on the side treated using fractional laser plus radiofrequency (30% excellent, 40% good, 22% sufficient, 8% insufficient)	Prolonged burning sensation above all on the laser + radiofrequency side, erythema and edema more on the laser side	
Guida, 2019 [19]	Atrophic surgical scars	9	33%	42.7 ±14.3 years (range between 35 and 65)	-	Retro	fractional picosecond-domain laser (PSL)	honeycombed pattern dermal papillae coarse collagen with collagen fibres showing a perpendicular orientation as compared to the major axis of the scar	After treatment micro-injuries, corresponding to black micro-holes surrounded by fringed borders within the surrounding tissue At 6-month, reticulated collagen fibers were observed to form a net. The increased brightness of the collagen fibres	6 months	Improvements in 66% of patients aesthetic satisfaction: very satisfied in 50% of cases, 40% satisfied and 10% not satisfied.	Mean pain score was 3.2 ± 1.5. Erythema, petechiae, crusts lasting 7–10 days while no hypo/hyperpigmentation was reported	Standardized photography can impair the evaluation of subtle changes in depth of treated scars, as previously reported for striae distensae. Therefore, aesthetic improvement can be too subtle to be captured in twodimensional digital images.
Guida, 2019 [20]	atrophic and hypertrophic surgical scars	16	50%	43.5 ± 13.5 years (range between 25 and 65)	-	Retro	fractionated 1064 nm wavelength, Nd:YAG PSL	At T0, epidermal features included the honeycombed pattern (80% of scars) while 20% of scars (all atrophic) showed cobblestone pattern Epidermal thickness was found to be related to the type of scar, with atrophic scars showing a mean thickness of 43 ± 10.3 μm and hypertrophic 58.5 ± 5.3 μm, *p* = 0.0005. In addition, at DEJ level, the majority of scars showed inconclusive features while 20% of scars (corresponding to the scars showing mottled pigmentation at epidermal level) had rare oval-shaped dermal papillae that can be observed in the context of lesional skin. Furthermore, coarse collagen or thick parallel collagen bundles, with a prevalent perpendicular orientation as compared to major axis of surgical scars, were observed at upper dermis	Overall, 100% of scar show honeycombed pattern. Significant reduction of epidermal thickness was observed for hypertrophic scars. A total of 75% of scars showed visible dermal papillae while at dermal level bright and thin reticulated collagen fibers were arranged to form a net in 95% of cases.	1 month	An overall improvement in 95% of patients. All four parameters of the VSS (pigmentation, vascularity, pli- ability, and height) were assessed, with a mean of 5.5 ± 1.4 at T0 and 3.1 ± 1 at T1, and a mean improvement of 2.4 ± 0.7, *p* = 0.000.	Three post-procedure events were reported: erythema, pete- chiae, and crusts.	
Guida, 2020 [21]	striae distensae	18	88%	31.6 ± 10.5 (range 18–54).	-	Retro	CO_2_ fractional	parallel collagen fibers were observed in 100% of cases and elongated parallel papillae in 94.1% of cases, while the neatwall was detected in 76.5% of the population.	Elongated parallel papillae, parallel collagen fibers, the neatwall significantly decreased after treatment. Interestingly, statistically significant reduction of parallel collagen fibers and neat-wall was observed in the group receiving >4 sessions of treatment, as compared to the group receiving ≤4 sessions.	4 weeks	overall improvement in 100% of cases, with 64.7% of patients very much improved assessed by SGAIS	local and self-limiting adverse events	a new confocal feature, called “neat-wall” was identified. “Neat-wall” corresponds to a distortion of the normal DEJ, visible only in mosaic images, similar to a well-demarcated wall separating regular areas of junction, which are formed by round papillae and fibrillar or reticular collagen
Fusano, 2021 [22]	striae distensae	27	100%	39.45 years (range: 25–55 years)	-	Retro	Picosecond laser	epidermal features of SD were represented by prevalent honeycomb or cobblestone pattern, focally less visible for the presence of atrophic and thin skin. At the dermal level, SD showed parallel reinforced collagen arranged in bundles, stretching and obliterating the dermal papillae, which appeared rare and oval-shaped in the context of lesional skin; hair follicles were not visible among the SD.	After 6 months from the last laser session (T1), the epidermis was entirely represented by a honeycomb or cobblestone pattern. At the dermal level, the hyperreflective reinforce of parallel collagen was no longer appreciable, and larger and more represented oval papillae appearence	6 months	An overall improvement in 81.4% of treated SD was revealed from PGAIS 6 months after the last laser session, while in 66.6% according to SGAIS (Table 1), with a significative difference between the investigators’ and subjects’ score (*p* = 0.04).	Two cases of purpura and micro-bleeding	
Pigmentary disorders													
Richtig, 2011 [23]	Solar Lentigines	12	100%	59.3 years, (range 49–69 years)	caucasian	Retro	Q-switched ruby	epidermal honeycomb pattern. At the DEJ, edged dermal papillae and policyclic papillary contours	After treatment disruption of the stratum corneum was confirmed by the presence of fine scales. The honeycomb pattern showed blurred epidermal intercellular connections, while dark structureless areas of different sizes and shapes were observed throughout the epidermis. Dermal papillae were markedly hyporeflective Ten days after treatment, stratum corneum was found still to be disrupted. Further extracellular deposits of melanin were observed. At the DEJ, non-edged dermal papillae were observed, in 9 cases containing a few melanophages. Bright reflective rims surrounding the dermal papillae were no longer observed at the DEJ.	30 min 10 days	-	-	Histological analysis of the biopsy tissue obtained from two patients revealed epidermal and dermal oedema, presenting as multiple cell debris-filled vacuoles of various sizes, some of them filled with cell debris, at the bases and tips of the rete ridge
Peng, 2021 [24]	Cafe’ Au Lait Macules (CALMs)	43	-	9.10 years.	Fitzpatrick skin Type III to IV	Retro	Q-switched alexandrite laser (QSAL)	length and density of papillae	CALMs with irregular border had significantly shorter rete pegs and less papillae on RCM compared with smooth border CAMLs and responded better to QSAL treatment (2.32 vs. 1.10)	3 months	We reported that CALMs with irregular borders respond better to QSAL treatment compared to those with smooth borders	Not found	Male patients have shown to achieve better clinical responses than females. The underlying mechanism might be related to different hormonal background
Xu, 2016 [26]	Infraorbital Dark Circles Color dark brown	30	100%	30.8 years (20–45 years)	Fitzpatrick III/IV	Open label study	fractional Q-switched ruby laser	greater melanin deposition in the upper dermis of the dark circles area than in cheekbone skin, although there was no significant difference in epidermal melanin density in the dark circles than in the cheekbone area	After eight treatment sessions, 6 of the 30 subjects (20%) obtained more than 75% clearance of the melanin deposition in the upper dermis (melanophages, personal comment), 15 (50%) obtained 50–74% clearance, 7 (23.33%) obtained 25–49% clearance, and only 2 obtained less than 25% clearance. Meanwhile, the melanin granules at the control site (the highest point of the cheekbone) remained at a low level during and after each treatment.	7 days 3 months 6 months	The melanin index indicated a sub-stantial decrease from 240.44 (baseline) to 194.56 (*p* < 0.05).	Transient erythema and slight edema were observed and usually resolved within 0.5 to 1 h after the procedure. No scarring, hyperpigmentation, hypopigmentation	
Xu, 2011 [25]	Infraorbital Dark Circles Color dark brown	30	100%	35.5 (range 20–42)	Fitzpatrick III/IV	Open label study	1064-nm Q-switched neodymiumdoped yttrium aluminium garnet (Nd:YAG)	greater melanin deposition in the upper dermis of the dark circles area than in cheekbone skin, although there was no significant difference in epidermal melanin density in the dark circles than in the cheekbone area	After eight treatment sessions, four of 30 subjects (13.3%) obtained more than 75% clearance of melanin deposition in the upper dermis (melanophages, personal comment), 16 (53.3%) obtained 50% to 74% clearance, eight (26.7%) obtained 25% to 49% clearance, and two obtained less than 25% clearance. The melanin granules in the control site (the highest point of the cheekbone) remained at a low level during and after each treatment.	6 months	The melanin index indicated a substantial decrease, from 225.84 at baseline to 182.65 (*p* = 0.05)	Transient erythema and slight edema were observed and usually resolved within 0.5 to 1 h after the procedure. No scarring, hyperpigmentation, hypopigmentation	
Jo, 2018 [27]	melasma	8	100%	49.00 ± 4.07 range 42–56 years	Fitzpatrick skin type III	Pro split face	Picosecond Alexandrite Laser (PAL) and Q-Switched (QS) Nd:YAG Laser 1064 nm	In the epidermis, evaluation of densely aggregated melanosomes in the keratinocytes of the spinous layer. Prominent dendritic melanocytes were present in the basal layer of the epidermis in three (37.5%) of eight subjects. At the DEJ level, papillary rings around the derma papillae composed of sequences of brighter structures and dendritic melanocytes. There were also melanophages in the papillary dermis. In surrounding normal skin, there was less melanin in the epidermis and DEJ than there was in the melasma lesions. The pigment intensity of each skin layer (spinous layer, basal layer, and papillary dermis) was investigated 1 and 24 h after treatment.	After PAL treatment, there was a decrease in melanin-induced reflectance in the spinous layer and basal layer. In contrast, treatment with QS Nd:YAG led to slight or non-significant improvement in the spinous layer and aggravated findings in the basal layer. After the treatments, a decrease of melanin-induced reflectance was shown in melasma lesion and surrounding normal skin. However, there was more improvement in the surrounding normal skin than in the melasma lesions. After 1 h of PAL or QS Nd:YAG treatment, 37.5% of subjects in both groups demonstrated melanocyte activation in the basal layer. One and two subjects showed melanocyte activation (dendritic) 24 h after PAL and QS Nd:YAG treatment, respectively. In addition, 37.5% of subjects showed perifollicular reflectance accentuation in the basal layer and upper dermis after treatment with QS Nd:YAG and PAL lasers, respectively	24 h	After a single treatment with either the picosecond alexandrite laser or the Q-switched Nd:YAG laser, both melanin-induced melanin index decreased, at 1 (19%) and 24 h (8%) for PAL and 1 (14%) and 24 h (6%) for QS. The melanin index decreased at 1 h after both treatments in surrounding normal skin.	N/A apart from aggravated findings in the basal layer after QS. However, short term evaluation (personal comment)	On the 30-fold magnified dermoscopic images with Vivacam, follicular plugs and hyperpigmented rings were observed in 6 (75%) and 4 (50%) of the melasma lesions from eight subjects, respectively (Figure 1). These hyperpigmented rings on dermoscopy were observed as hyper-reflectant rings on RCM in 4 (50%) of the melasma lesions
Longo, 2014 [28]	melasma	8	100%	mean age 36.7 (31–41)	Fitzpatrick skin type III	Retro	Q-Switched (QS) Nd:YAG Laser 1064 nm	The epidermis showed a honeycombed pattern, and in three cases (37.5%). In one case, bright dendritic cells located around hair follicles were visualized. At DEJ, all cases showed bright polycyclic contours and bright hair follicles/rings at DEJ level. In the upper dermis, no bright cells referable to melanophages or other structures (inflammatory cells, melanin particles) were observed.	After nine laser sessions, no mottled pigmentation or polycyclic papillary contours were observed. The presence of dendritic-shaped cells was noted in three cases at epidermal and DEJ levels. However, after a follow-up of 3 months following the ninth laser session, the cases presenting dendritic cells had an early relapse of melasma.	unclear	Clinically, all patients improved showing a statistically significant decrease of the MASI score (*p* < 0.001).	Erythema, edema, and scaling were reported but their severity was mild and well tolerated. In particular, erythema and edema disappeared in a few hours, while fine scaling lasted from 2 to 3 days and was minimized by common hydrating creams.	
Vascular disorders													
Fu, 2019 [30]	Port wine stain	42	59%	from 1 to 48 years average age of 14.3 years.	-	Pro	585/1064 nm laser treatment	blood vessels in the papillary dermis. average blood vessel depth, density, flow	PWS blood vessel flow, diameter, and depth, but not density, are the key factors contributing to the laser-resistance. In our study, we showed that the average diameter of laser-responsive facial PWS blood vessels decreased from 84.36 to 49.04 μm after laser treatment, suggesting that the remaining PWS blood vessels (diameters < 49.04 μm) were more resistant to laser treatment. Results have shown that laser-resistant facial PWS blood vessels had significantly higher blood flow, larger diameters, and were located deeper in the skin when compared with responsive PWS on the face	4 weeks	Three out of 33 subjects with facial PWS showed a complete response, 12 showed manifested responses, 13 showed improvements, and 5 showed an ineffective response, as estimated from pictures. Overall response: 84.85%		44.4% PWS on the extremities (four out of nine subjects) were laser-resistant, which was significantly higher (*p* < 0.001) when compared with those PWS on the face (15.2%, 5 out of 33 subjects). Laser-resistant PWS blood vessels had significantly higher blood flow, larger diameters, and were located deeper in the skin. RCM can be a valuable tool for a prognostic evaluation on laser-resistant lesions before treatment, thereby providing guidance for tailored laser treatment protocols
Ren, 2014 [29]	Port-wine stains	11	45%	24.82 ± 9.82 years	-	Pro	595 nm Pulsed Dye Laser	Blood vessels diameter and blood vessels density RCM imaging at the depth of 100 and 150 μm showed greater vessel diameter before treatment and the vessels tended to increase in caliber with increasing depth,	After treatment, both diameter and density of blood vessel decreased significantly as compared with those before treatment in the same pulse-duration groups (*p* < 0.05). There was significant difference between 1.5 ms pulse-duration group and other pulse duration groups in reducing blood vessel diameter at the depth of 150 μm (*p* < 0.05), while no signifi cant difference among each pulse-duration groups at the depth of 100 μm (*p* > 0.05).	2 months	Overall, clearance was excellent in 27.28% of patients, good in 20.45%, fair in 36.36%, and poor in 15.91%. The PDL treatment exhibited increasing clearance with reducing pulse duration.		
Other applications													
Kim, 2022 [31]	Wound healing after laser treatment f healthy skin inner arms	8	87.5%	24 and 48 years (median 39.62)	Fitzpatrick Skin Types II to IV	Pro	fractional CO_2_ laser	The decrements of the ablative zone were evaluated at the level of the stratum spinosum and stratum basale. The ablative zone was evaluated using a six point scale based on the percentage change in area in proportion to the baseline (D3) (i.e., 125%, slightly worse [slightly expanded]; 100%, no change; 75%, slightly improved [slightly decreased]; 50%, moderately improved [moderately decreased]; 25%, significantly improved [significantly decreased]; and 0%, completely improved [completely decreased]). areas with a whitish ring exhibiting high reflectance in stratum spinosus, basale and papillary dermis.	The ablative zone of the stratum spinosum showed a progressive reduction at 14-day after treatment and complete resolution at day-28. The ablative zone was smaller at the basal layer as compared to thte stratum spinosum. Thermally modified zones, surrounding the ablative zones, were observed at different time periods. From day3 to day14, the thermally modified zone was visualized as a highly refractile ring. At day28, even after epidermal regeneration was completed, a thermally modified zone was observable in some subjects. Bright and fine fibers corresponding to newly formed collagen throughout the papillary dermis were also visible. Two months after laser application, a fine and bright collagen network was consistently observed.	Up to 2 months	-	All subjects exhibited mild erythema and edema on the surface of the test skin directly after fractional CO_2_ laser application. By day-14, all the crusts had fallen and the skin surface was completely healed on macroscopic examination.	The MTZ comprised an ablative microchannel and a surrounding thermally modified zone, which included the coagulated and thermally denatured zones around the ablative zone.
Aghassi 2000 [32]	sebaceous hyperplasia	10 (29 lesions)	66%	30 to 57 years	Fitzpatrick skin type I–III.	Retro	585 nm pulsed-dye laser	Dilated sebaceous duct opening directly to the epidermal surface and containing a plug of keratin and sebum. At the papillary dermis, a “crown” of blood vessels surrounds the sebaceous duct. Deeper in the dermis, dilated blood vessels could be found in the vicinity of the duct. The enlarged sebaceous lobules were too deep to be visualized by RCM.	Several minutes after pulsed-dye laser treatment, the vessels surrounding the sebaceous duct were replaced by amorphous, refractile cords of coagulated material. Follow-up images obtained at 2 weeks, 4 weeks, and 8 weeks after treatment were not reproducibly or significantly different from pretreatment images, with the exception of 3 patients who demonstrated a temporary absence of keratinocytes overlying the treated lesion at the 2-week follow-up.	Up to 8 weeks	complete disappearance of 28% of lesions. decrease in diameter in 66%, and flattening in 93%	Only one cutaneous depression remained at the 8- week follow-up. Eight lesions (28%) recrudesced after initial involution, and 2 of these (7%) regained their original sizes. No frank scarring or pigmentary side effects were seen.	

Retro = retrospective; Pro = prospective.

## Data Availability

Not applicable.
